# Primitive Neuroectodermal Tumor of the Liver: A Case Report

**DOI:** 10.1155/2011/748194

**Published:** 2011-12-27

**Authors:** Eduardo Cambruzzi, Enilde Eloena Guerra, Hamilton Cardoso Hilgert, Herbert Jorge Schmitz, Vinícius Lopes Silva, Daniel Marini Milani, Ricardo Pedrini Cruz, Raul Pruinelli

**Affiliations:** ^1^Department of Pathology, Nossa Senhora da Conceição Hospital, Porto Alegre, Rio Grande do Sul, Brazil; ^2^Department of Surgery, Nossa Senhora da Conceição Hospital, Porto Alegre, Rio Grande do Sul, Brazil

## Abstract

Primary liver sarcomas represent a rare group of neoplasias, with angiosarcoma being the most common histological type. Primitive neuroectodermal tumor (PNET) represents a high malignant neoplasia that usually affects the central nervous system and soft tissues. An 18-year-old male patient was admitted with clinical complains of pain in the right upper abdominal quadrant. The clinical evaluation revealed a solid mass in the right hepatic lobe. On the gross examination of the resected liver specimen, the right lobe of the liver was replaced by a yellow-red solid mass measuring 21 cm in its largest dimension. On the histopathology, a tumor composed of small round blue cells with little cytoplasm and round nuclei was identified. The lesion revealed positive immunoexpression for vimentin and CD99 and negative immunostaining for desmin, CD45, cytokeratin, and neuroblastoma protein, suggesting, then, the diagnosis of PNET. Although it is an unusual tumor, it should be considered in the differential diagnosis of liver masses, especially in young patients.

## 1. Introduction

Liver neoplastic lesions include many different histological types of primary benign and malignant masses and high rates of metastatic processes. Primary tumors can be solid or cystic and can arise from hepatocyte, bile duct epithelium, neuroendocrine cells, mesenchymal cells, and, rarely, from heterotopic tissues [1, 2]. Hepatoblastoma and mesenchymal hamartoma are usually found in the pediatric population. Hepatocellular carcinoma represent the single most common histologic type of malignant epithelial tumors of the liver (about 85–90%), being frequently associated to cirrhosis and chronic viral hepatitis. Primary hepatic sarcomas are exceedingly unusual, accounting for only 1% to 2% of all malignant tumors arising in the liver, with angiosarcoma and undifferentiated sarcoma being the most common histologic types [1, 2].

 PNET represents a family of tumors which shows varying degrees of neuronal differentiation with an Ewing's sarcoma gene rearrangement, most often as a consequence of a *t* (11; 22) (q24; q12) chromosomal translocation. PNET is a highly malignant neoplasm most commonly involving the central nervous and skeletal system, and it is composed of small, round, uniform cells. Because of the undifferentiated appearance of the tumor cells, it looks as if the original cell might be an undifferentiated mesenchymal cell [3–5]. Herein, the authors report a case of PNET arising in the liver and review the morphologic and immunohistochemical findings of this tumor in a rare topography.

## 2. Case Report

**Figure 1:**
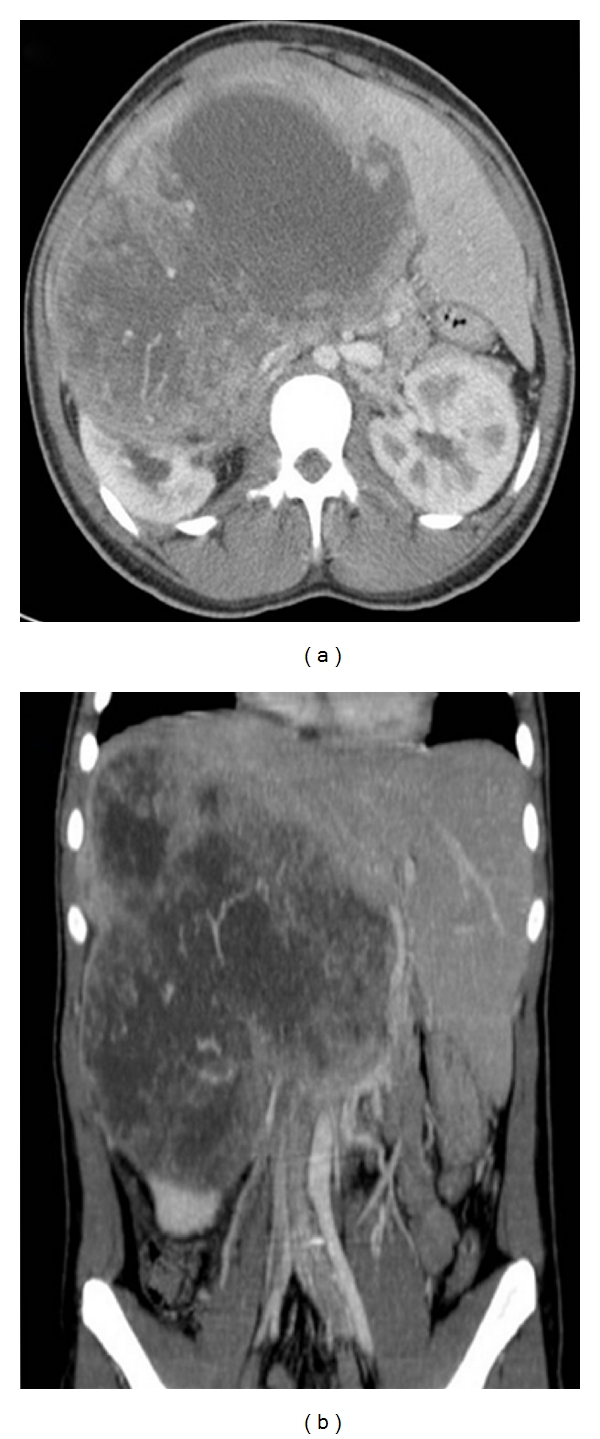
Preoperative CT scan showing a large, solid mass in the liver.

An 18-year-old male African descent patient was admitted complaining of pain in the right upper quadrant of the abdominal region for the last 3 months. The physical examination revealed a firm, solid mass with an irregular lower border and mild tenderness in the liver topography. Abdominal ultrasonography and computed tomography (CT) scans showed a solid mass in the right hepatic lobe, measuring 21 cm in its largest diameter, with possible colon and right kidney invasion, and vena cava compression (Figure 1). Preoperative right hepatic artery embolization was performed. In follow-up CT, areas of partial necrosis and no tumor size regression were found. The patient underwent exploratory laparotomy. The right lobe of the liver was partially replaced by a yellow-purple-red solid tumor, which was in continuity with the right kidney and hepatic colon flexure. There were no signs of peritoneal disease and no evidence of metastasis in liver segments 1 to 3. A right extended hepatectomy with en bloc resection of the right kidney, gallbladder, and partial colectomy (hepatic colon flexure) with primary anastomosis was performed.

**Figure 2:**
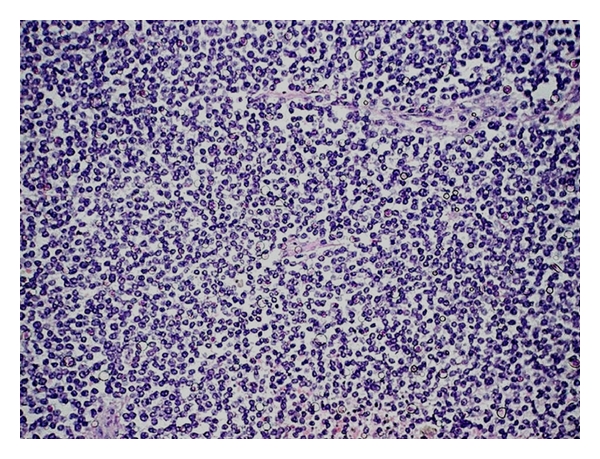
PNET arising in the liver: diffuse sheet of uniform small round cells in a lobulated growth pattern, HE, 200x.

**Figure 3:**
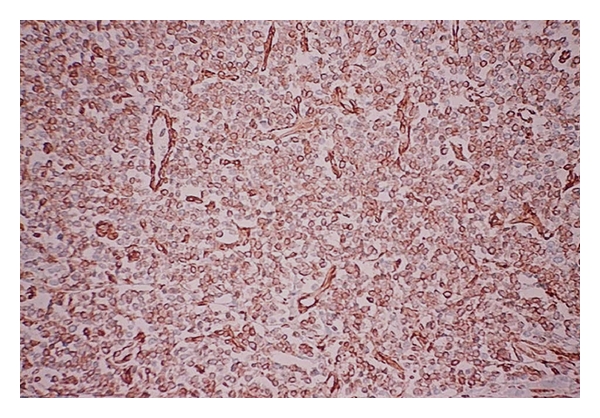
PNET arising in the liver: positive immunoexpression for vimentin, streptavidin-biotin, 200x.

**Figure 4:**
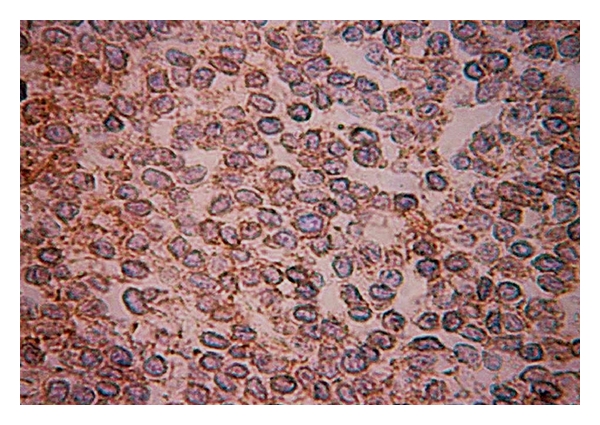
PNET arising in the liver: the tumor cells show immunoreactivity for CD99, streptavidin-biotin, 200x.

 The surgical specimen consisted of a portion of the liver, of the gallbladder, a segment of the colon, and of the right kidney, previously fixed in formalin, measuring 30 cm × 25 cm × 13.5 cm, and weighing 4313 g. On cut surface, the hepatic parenchyma was replaced by a yellowish-gray, multilobulated, soft tumor, with yellow central areas, that measured 21 cm in its largest diameter. The lesion was adhered to the colon and kidney, but without clear signs of invasion. On hematoxylin-eosin staining, a neoplastic process composed of sheets or lobules of small round cells, with little cytoplasm, and darkly staining, round or oval nuclei, was identified (Figure 2). The lesion also showed areas of necrosis, high mitotic index, and rare rosettes. The immunohistochemical study of lesion revealed positive expression for vimentin (Vim 3B4—Figure 3) and CD99 (12E7—Figure 4), and negative immunostaining for desmin (D33), cytokeratin (AE1/AE3), and neuroblastoma protein (NB84). The liver parenchyma adjacent to the tumor had a normal histologic appearance. The morphology of the hepatic lesion associated with the findings of the immunohistochemical study was consistent with PNET. One month after surgery, venous thrombosis extending from the iliac vessels to the right atrium and multiple metastatic implants in the lungs were identified in thoracic CT. The patient had a sudden death due to massive pulmonary thromboembolism. At this time, it was not possible to perform genetic studies to establish the lineage of the neoplasm. 

## 3. Discussion

PNETs are small round cell tumors that belong to the Ewing's sarcoma family of tumors. These tumors are divided in two main categories: central (derived from the neural tube) and peripheral (outside the central nervous system) [6–10]. PNETs were first described by Stout in 1918 [7, 10, 11] and represent less than 1% of all sarcomas [7, 12]. These sarcomas predominantly affect bones and deep soft tissue and rarely affect visceral organs. PNET can be found in the thorax (44%), abdomen and pelvis (44%), extremities (20%), and 6% in the head and neck areas [9, 12]. Visceral primary commitment has been described with increasing frequency in the pancreas, vagina, stomach, small bowel, ovaries, esophagus, and kidney [5–10]. 

The gross appearance of the tumor varies. In general, it is multilobulated, soft, and friable; it rarely exceeds 10 cm in its largest dimension. Its cut surface has a gray-yellow or gray-tan appearance, often with large areas of necrosis, cyst formation, or hemorrhage. Despite the extensive necrosis, calcification is rare [4, 5, 13]. 

At microscopy, the typical PNET shows a predominantly lobular growth pattern, with little or no stroma. It is composed of poorly differentiated small round cells containing darkly staining, round or oval nuclei. The pale eosinophilic cytoplasm is indistinct except in areas where the cells are more mature and the elongated hair-like cytoplasmic extensions coalesce to form rosettes. Most of the rosettes are similar to those seen in neuroblastomas and contain a central solid core of neurofibrillary material. Rarely do the rosettes contain a central lumen or vesicle. Some tumors are composed of cords or trabeculae of small round cells that can be mistaken by a carcinoid tumor or a small-cell undifferentiated carcinoma. The presence of areas of necrosis is a usual finding. There are uncommon tumors with glial, ependymal, cartilaginous, or epithelial differentiation. Some areas can resemble a fibrosarcoma or a malignant schwannoma [4, 5, 13, 14]. The ultrastructural features include the presence of elongated cell processes that interdigitate with each other and contain small dense-core granules (neurosecretory granules) that measure 50–100 nm and occasionally contain microtubules [14, 15]. On immunostains, many PNETs are usually positive for CD99, neuron-specific enolase, Leu-7, S-100, and synaptophysin, and negative for desmin and Myogenin/MyoD1. Some cases are positive for cytokeratin [3–5, 14–16]. PNET characteristically exhibits a neural phenotype, expressing the MIC2-protein (CD99), and display the *t* (11; 22) (q24; q12) chromosomal translocation in about 85%–95% of the cases [3, 7, 8, 17–22].

The differential diagnosis includes neuroblastoma (positive for NB84), desmoplastic small round cell tumor, mesenchymal chondrosarcoma, alveolar rhabdomyosarcoma, non-Hodgkin's lymphoma, metastatic pulmonary small-cell carcinoma, cutaneous neuroendocrine carcinoma, small-cell osteosarcoma, and poorly differentiated synovial sarcoma [3–5, 13, 18–20, 22].

 Treatment with surgery alone for extraskeletal PNET is considered insufficient, and multimodal treatment with chemotherapy and radiotherapy is frequently performed. Despite the multimodal treatment, most patients show a rapid dissemination of the disease. Key prognostic factors that adversely influence the outcome are the presence of metastatic disease at the time of the initial diagnosis, large tumor size, extensive necrosis, central axis tumors, and poor response to initial chemotherapy. Patients with type 1 EWS/FLI fusion transcripts appear to have longer disease-free survival than those with other fusion transcript types [3, 5, 6, 19–21].

## 4. Conclusion

PNETs are usually aggressive small blue cell tumors arising in the central nervous or skeletal system. To the best of our knowledge, this is the third case report of this tumor arising in liver parenchyma. Although it is an unusual liver tumor associated with poor prognosis, PNET should be considered in the differential diagnosis of liver masses, especially in young patients.
